# Distinct patterns of gene expression in the medial preoptic area are related to gregarious singing behavior in European starlings (*Sturnus vulgaris*)

**DOI:** 10.1186/s12868-023-00813-4

**Published:** 2023-08-03

**Authors:** Brandon J. Polzin, Sharon A. Stevenson, Stephen C. Gammie, Lauren V. Riters

**Affiliations:** Department of Integrative Biology, University of Wisconsin- Madison, Madison, WI USA

**Keywords:** Songbirds, Gregarious, Bioinformatics, Flocking, Co-expression network, Glutamate, Neurotranscriptome, Preoptic area, Reward, Communication

## Abstract

**Background:**

Song performed in flocks by European starlings (*Sturnus vulgaris*), referred to here as gregarious song, is a non-sexual, social behavior performed by adult birds. Gregarious song is thought to be an intrinsically reinforced behavior facilitated by a low-stress, positive affective state that increases social cohesion within a flock. The medial preoptic area (mPOA) is a region known to have a role in the production of gregarious song. However, the neurochemical systems that potentially act within this region to regulate song remain largely unexplored. In this study, we used RNA sequencing to characterize patterns of gene expression in the mPOA of male and female starlings singing gregarious song to identify possibly novel neurotransmitter, neuromodulator, and hormonal pathways that may be involved in the production of gregarious song.

**Results:**

Differential gene expression analysis and rank rank hypergeometric analysis indicated that dopaminergic, cholinergic, and GABAergic systems were associated with the production of gregarious song, with multiple receptor genes (e.g., DRD2, DRD5, CHRM4, GABRD) upregulated in the mPOA of starlings who sang at high rates. Additionally, co-expression network analyses identified co-expressing gene clusters of glutamate signaling-related genes associated with song. One of these clusters contained five glutamate receptor genes and two glutamate scaffolding genes and was significantly enriched for genetic pathways involved in neurodevelopmental disorders associated with social deficits in humans. Two of these genes, GRIN1 and SHANK2, were positively correlated with performance of gregarious song.

**Conclusions:**

This work provides new insights into the role of the mPOA in non-sexual, gregarious song in starlings and highlights candidate genes that may play a role in gregarious social interactions across vertebrates. The provided data will also allow other researchers to compare across species to identify conserved systems that regulate social behavior.

**Supplementary Information:**

The online version contains supplementary material available at 10.1186/s12868-023-00813-4.

## Background

Birdsong has been well studied in males for its role in mate attraction and territory defense [1]. However, some species, such as European Starlings (*Sturnus vulgaris*), also sing at high rates outside of the breeding context. In starlings, song in a non-breeding context is produced at high rates by both males and females in flocks and is considered essential for birds to develop and practice songs that are later used in breeding contexts [2, 3]. Song in a non-breeding context is also considered important for maintaining flock cohesion [4]. Although much is known about the neural regulation of song learning and the production of male courtship songs, relatively little is known about the neural mechanisms that facilitate and maintain song in gregarious contexts, which will be referred to here as gregarious song.

Most of the existing literature has centered around the medial preoptic area, commonly abbreviated POM in birds and mPOA in mammals. Here we refer to the structure as the mPOA to indicate that findings may generalize beyond birds, as highlighted below. The mPOA is a highly conserved structure that is well known for its role in sexually-motivated behaviors but more recently has also been shown to be important in the control of non-sexual social behaviors, including gregarious song [5]. Lesions of the mPOA tend to increase gregarious song, and the presence and activation of mu opioid receptors (inhibitory receptors) in the mPOA stimulates song, suggesting an inhibitory role for the mPOA in gregarious song [6–8]. Studies show that a positive affective state associated with gregarious song correlates positively with the expression of opioid-related genes in the mPOA [9], and downregulation of mu opioid receptors in the mPOA reduces singing and breaks the link between positive affect and singing behavior [8]. Results of a recent study also show that mu opioid receptor stimulation in the mPOA stimulates components of song (i.e., introductory whistles), reduces a potential sign of stress (i.e., beak wiping), and induces reward (i.e., a conditioned place preference), suggesting that opioid activity in the mPOA may both stimulate and reward gregarious singing behavior [10]. Although opioids have been best studied, studies focused on other candidate modulators of motivation and reward also implicate dopamine D1 receptors [11], endocannabinoids [12], and possibly alpha 2 norepinephrine receptors [13] in the mPOA in gregarious singing behavior; however, these studies are limited by a focus on previously-known candidate genes. There are likely many more neurochemicals and signaling cascades within the mPOA driving this behavior that remain to be uncovered.

The mPOA is part of a social behavior network that is highly conserved across vertebrates [14]. We have already found that the role of mu opioid receptors in the mPOA identified by studies of gregarious song [8–10, 15] extends to other non-sexual rewarding social behaviors in rodents (i.e., rat social play) [16]. Thus, identifying novel neuromodulators within the mPOA associated with gregarious song is expected to reveal critical, conserved mechanisms that regulate other important non-sexual social behaviors across vertebrates.

This study aimed to uncover potentially novel neurotransmitters, neuromodulators, and hormone pathways within the mPOA associated with gregarious song. To do this, we observed male and female European starlings singing in flocks, then performed RNA-sequencing (RNA-seq) in punches from the mPOA to compare relative gene expression between birds that sang at low and high rates. We applied multiple bioinformatics approaches to gain insight into new candidate systems associated with gregarious song. These ranged from hypergeometric analysis to compare expression profiles across sexes, to using multiple methods of co-expression analyses to find likely “hub” genes associated with differences in gregarious song production that likely regulate other prosocial behaviors in vertebrates.

## Materials and methods

### Animals

Twenty-two adult European starlings (11 males [with testes], 11 females [with ovaries]) were used in this study. All starlings were trapped from the wild on a farm on the west side of Madison, Wisconsin in October of 2019. No specific permissions were necessary for capturing starlings from the wild. They are considered invasive and are not endangered, protected, or covered under the Migratory Bird Treaty Act in the United States. After capture, all birds were housed in indoor stainless-steel cages at the University of Wisconsin-Madison. Birds were put on an 18L:6D cycle (lights on at 6:00am) for at least 6 weeks to induce a state of “photorefractoriness”. This is a physiological state in early fall in which starlings begin to sing in large, mixed-sex flocks [17]. All studies were approved by the University of Wisconsin Institutional Animal Care and Use Committee and in accordance with the Guidelines of the National Institutes of Health.

### Behavioral observations

Focal birds were moved to indoor aviaries (2.12 × 2.4 × 1.98 m) with natural tree branches and given *ad libitum* access to food, drinking water, and bathing water. Birds were in mixed-sex flocks of 8 birds (4 males, 4 females). Talk radio was played during daylight hours to acclimate birds to voices and extraneous noise. All observations were conducted from September 2020-November 2020 by the same observer behind a one-way mirror.

Observations began once 4 or more birds were singing within an aviary. For each observation period, a male or female focal bird was selected based on the rate of song observed during earlier observation periods. This strategy was employed to ensure a broad representation of song rate production in the birds included in our study. Focal observations were performed on the targeted bird in the aviary for 20 min a day, 5 days in a row. All observations took place between 0900 and 1200 h each day. Immediately preceding each observation, a recording of starling song was played for 5 min to instigate singing from the aviary. Then song was measured by a point sampling method. Every 20 s during the observation period, the observer was cued by a quiet vibration of a smartwatch and recorded a tally if the bird was singing. The quantitative measure of the song was the number of tallies that were recorded over 5 days (maximum of 300 possible). The observer also continuously recorded locomotion, feeding, drinking, and agonistic interactions. All behavioral data are available in Supplementary Material S1.

Thirty minutes after the final observation period, the bird was caught by net in the aviary and sacrificed by rapid decapitation. We waited 30 min as mRNA for immediate early gene expression tends to peak 30 min after stimulation [18]; however, for this study we were interested in constitutive gene expression associated with the propensity to sing. Thus, we examined relationships between patterns of gene expression and song measurements summed across the 5 test days. The brain was flash-frozen using dry ice and stored in -80 °C freezer until observations of the last birds were completed in November. A replacement bird was then moved from the cages in the animal facilities to the aviary to maintain a total of 8 birds in an observation aviary. Once 4 or more birds were singing again, the above protocol was repeated until 11 male brains and 11 female brains were collected.

### Tissue collection

**Fig. 1:**
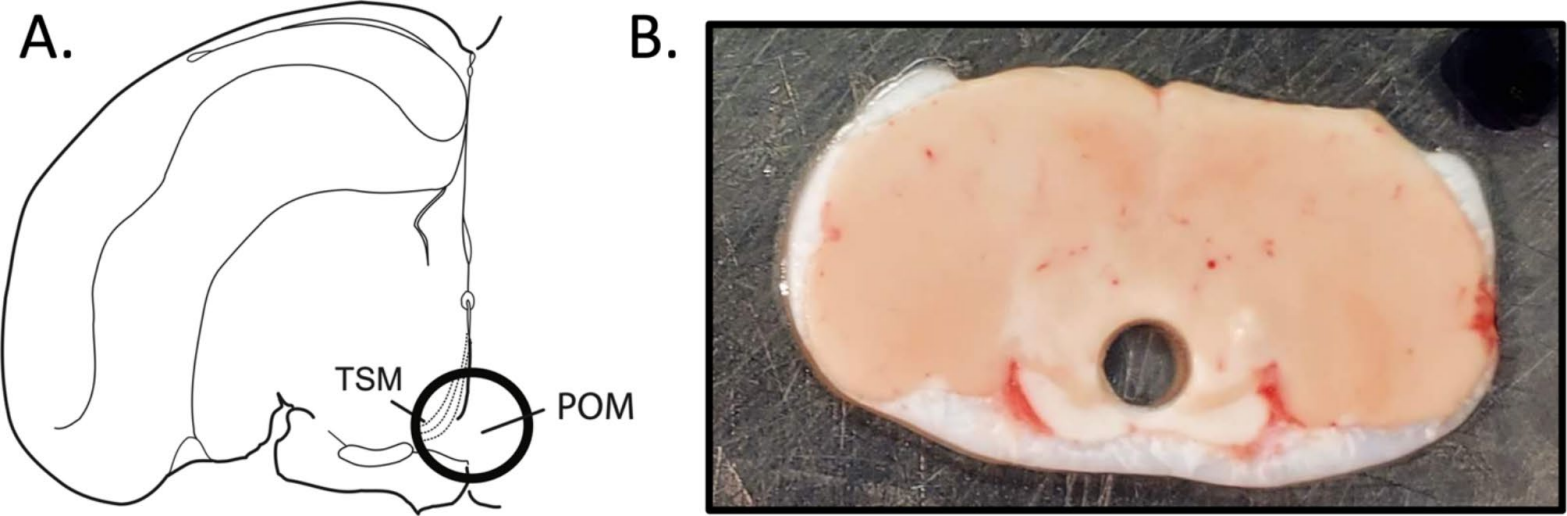
** A)** Illustration created in-house of one hemisphere of the starling brain showing the location of the mPOA where punches for RNA-sequencing were taken. Punches (2 mm diameter) were taken slightly caudal to the end of the visible tractus septomesensephalicus (TSM) and rostral to the anterior commissure, in the center of the brain surrounding the 3rd ventricle. **B)** Example image of one of the mPOA punch areas

Whole brains were cut in 200 μm sections in the coronal plane using a cryostat at -15 °C and mounted onto glass slides. Two mm punches (Fine Science Tools Sample Corer, 2 mm, Item no. 18035-02) for the mPOA were taken from a single slice in each bird caudal to the end of the visible tractus septomesencephalicus and rostral to the anterior commissure, in the center of the brain surrounding the 3rd ventricle (Fig. 1) then stored at -80 °C. An example photo of an mPOA punch can be found in Fig. 1.

### RNA processing and RNA-seq

All sequencing was performed by the University of Wisconsin-Madison Biotechnology Center’s Next Generation Sequencing Facility. The comprehensive methods used for total RNA verification and processing for RNA sequencing, which align with our prior work [20], are as follows. Total RNA was extracted with the Aurum Total RNA Fatty and Fibrous Tissue Kit (Bio-Rad, Hercules, California). A NanoDrop One Spectrophotometer was used to verify RNA purity and an Aglient 2100 Bioanalyzer was used to verify RNA integrity. The purified RNA was stored at -80 °C until it was sent for sequencing. The samples met Illumina input guidelines, and were then prepared using the Illumina® TruSeq® Stranded mRNA Sample Preparation kit (Illumina Inc., San Diego, CA, USA). The mRNA was purified from 150ng total RNA using poly-T oligo-attached magnetic beads for each library preparation. Afterwards, each poly-A enriched sample was fragmented using divalent cations. SuperScript II Reverse Transcriptase (Invitrogen, Carlsbad, CA, USA) was then used to synthesize fragmented RNA into double-stranded cDNA. Random primers were used for first strand cDNA synthesis and then second strand cDNA synthesis using DNA Polymerase I. RNase H was used to treat the double-stranded cDNA to remove mRNA, and was then purified by paramagnetic beads (Agencourt AMPure XP beads, Beckman Coulter). The cDNA products were exposed to Klenow DNA Polymerase, which attached an ‘A’ base (Adenine) to the 3’ end of the blunt DNA fragments. DNA fragments were ligated to Illumina unique dual adapters with a single Thymine overhang on the 3’ end. This as amplified in a Linker Mediated PCR reaction for 12 cycles using Phusion™ DNA Polymerase and then purified using paramagnetic beads. The finished libraries were assessed for quality using an Agilent HS DNA chip (Agilent Technologies, Santa Clara, CA, USA) and assessed for quantity using a Qubit® dsDNA HS Assay Kit (Invitrogen, Carlsbad, CA, USA). Libraries were standardized on 2 nM and paired-end 2 × 150 base pair sequencing was performed using an Illumina NovaSeq6000 sequencer.

All samples (for 11 males and 11 females) had sufficient raw reads, so all were included in downstream analyses. For all analyses we used the 2021 zebra finch (*Taeniopygia guttata*) National Center for Biotechnology Information (NCBI) Release (2021-ZF) (GCA_003957565.4) for gene annotation. We also examined the slightly older 2019 zebra finch (*Taeniopygia guttata*) NCBI Release (GCA_003957565.2), and the European starling (*Sturnus vulgaris*) 2015 NCBI Release (GCA_001447265.1), but found results to be highly similar, so the 2021 annotation was used for all analyses because it is the most complete in terms of annotated genes. RNA expression was normalized using RSEM [21], a method allowing for improved RNA-seq assembly and quantification without a fully sequenced genome. The RSEM values for 2021 zebra finch and 2015 starling annotations can be found in Supplementary Material S1.

### Differential gene expression analysis and rank-rank hypergeometric analysis

All analyses below were performed in R-Studio (v.1.3) with R (v.4.1.1).

**Fig. 2:**
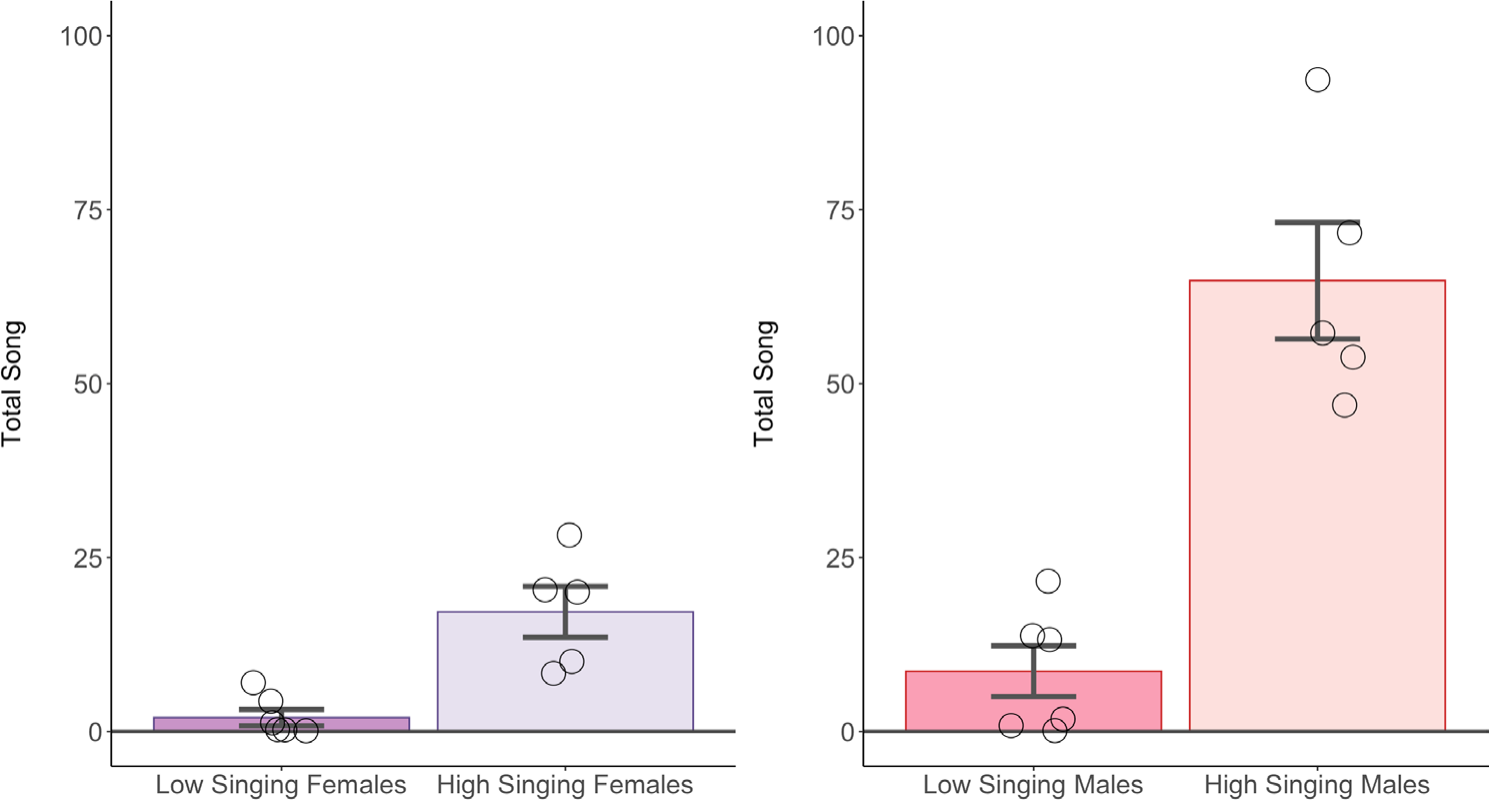
Shows total song of individual birds in the 4 groups used in differential gene expression and rank-rank hypergeometric overlap analysis. Birds were separated by sex, and a median split based on the total point-sampled song over 5 days was performed among males and females. Both low-singing and high-singing males sang more than their respective singing conditions in females

Samples were separated by sex, then further separated by performing a median split based on song, creating a “low-singing” and “high-singing” group of birds within each sex (low-singing males; n = 6, high-singing males; n = 5, low-singing females; n = 6, high-singing females; n = 5) (Fig. 2). We then performed a differential gene expression (DGE) analysis between the high and low singing groups for each sex (totaling 2 analyses) using the EdgeR Bioconductor Package, v. 3.1.2 [22]. Although this work concentrates on song-associated gene-expression changes, we also performed DGE analysis comparing the mPOA of both males and females to provide insight on sex differences in the mPOA in a non-breeding context.

Following this, rank-rank hypergeometric overlap (RRHO) analysis [23] was performed to compare patterns of gene expression direction across sexes. RRHO generated heatmaps that allowed us to see both congruent (same direction) and discordant (opposite direction) relationships across each sex’s expression profile (e.g., which genes were consistently upregulated in both high-singing males and high-singing females, and vice versa).

### WGCNA

We used the common systems biology approach of weighted gene co-expression network analysis (WGCNA) to create single scale gene co-expression networks and gene modules from gene expression data sets [24]. RRHO showed there was statistically significantly high overlap between the top 800 most differentially expressed genes between males and females, so the sexes were combined for network analyses. Before network analysis began, genes with low expression across samples within each dataset were removed, transitioning from 21,721 original genes to 9,648 filtered genes.

For the first analysis, a weighted network of genes (nodes) and their expression correlations (edges) were generated, with correlations raised to a soft-thresholding power β of 7. In this study, the β values for all WGCNA networks were chosen following the guidelines put forth by the authors of WGCNA [25]. We identified the β value as the point in network topology analysis at which any increase in β would lead to only marginal improvements in the fit of the scale-free topology model. Initial parameters were as follows: unsupervised hierarchical clustering was used; signed mode; minimum module size = 30; deepSplit = 2; MergeCutHeight = 0.3. Due to our primary interest in transcriptomic differences between the two groups of low- and high- singing birds, we also performed WGCNA’s module preservation analysis to compare network connectivity between the two groups. Two separate networks were created, one with only low-singers and one with only high-singers. Additionally, genes not expressed in both low- and high- singing datasets were removed to ensure networks were comparable for module preservation analysis. WGCNA was performed on each dataset of 8,967 genes.

Two weighted networks of genes (nodes) and their expression correlations (edges) were generated, with correlations raised to a soft thresholding power β of 9 and all other parameters the same as above. We then used the modulePreservation WGCNA function to identify which modules of co-expressing genes showed no evidence of being preserved between the two analyses, implying possible functional differences between those modules [26]. Analysis was performed with parallelization disabled and nPermutations = 200.

Statistics for module preservation were used to determine if a specific module defined in the low-singing dataset (reference dataset) could also be found in the high-singing dataset (test dataset). Results are reported as a summary of Z-statistics (Zsummary). In short, the Zsummary is a composite measure of the density-based and connectivity-based preservation statistics generated by WGCNA’s permutation test. For each module, four connectivity-based and three density-based preservation statistics are generated, each with its corresponding Z-statistic. These Z-statistics follow a normal distribution if the module is not preserved; a higher Z-statistic value indicates a stronger likelihood that the preservation statistic surpasses what would be expected by chance. The Zsummary is calculated by taking the median of all the connectivity and density-based Z-statistics, summing them, and dividing by two. For a detailed mathematical explanation of this methodology, please refer to the original publication [26]. A Zsummary of > 10 is indicative of high module preservation between the two networks (e.g., blue module in low-singers is almost indistinguishable from blue module in high-singers), Zsummary between 2 and 10 represents moderate module preservation, and Zsummary < 2 indicates that there is almost no preservation between modules (e.g. genes in blue module in high-singers are almost entirely different from those in the blue module of low-singers). The hub genes in each module were chosen using the chooseTopHubInEachModule WGCNA function.

### MEGENA & CTA

Multiscale embedded gene co-expression network analysis (MEGENA) is a hierarchical clustering framework that allows for the construction of gene co-expression networks and gene modules using planar-filtered networks and multiscale clusters [27]. This method identifies nested groups of highly correlated genes from a given tissue sample. MEGENA, unlike WGCNA, creates a multi-scale network, allowing one single network to include multiple variations of gene interactions (i.e., one gene can be found in multiple modules).

Our dataset included all animals, and genes were filtered out for low expression. 11,447 genes were processed by the MEGENA package in R. Parallelization (nCores = 8) was turned on to increase speed of analysis. The FDR cutoff, module significance p-value, and connectivity significance p-value were all set at 0.05. The number of permutations for calculating FDR was set to 10 and the number of permutations for calculating the connectivity significance p-value was set to 100.

To link MEGENA gene modules to song, cluster-trait association (CTA) analysis was performed. CTA allows us to correlate expression patterns for groups of genes with performed behaviors. A principal components analysis was run on each of the modules using the ModulePrinComps function in the MEGENA package [27], and the first principal component of the expression levels of all genes within the module was used as a new variable, known as an eigengene (see Supplementary Material S4). This gives each gene module a representation as a continuous variable for each animal and allows for behaviors to be correlated with co-expressing groups of genes that are likely functionally connected. Pearson correlations were performed to compare module eigengenes with singing condition.

### Enrichment analysis

Enrichment analysis was performed on gene sets produced by DGE and RRHO and on modules created by WGCNA and MEGENA. This method allowed us to input a custom list of genes and determine if those genes are over-represented in large gene sets with previously known functions. If a custom set of genes is overrepresented in a functionally known gene set, it is notated as “significantly enriched”. These previously identified gene sets include molecular pathways, drug actions, diseases, and many others. Enrichment of all MEGENA modules was performed using the moduleGO function in the DGCA package in R for general exploration [28]. Follow up enrichment tools for investigating our lists of interests included ToppCluster [29], GeneOntology [30, 31], and STRING [32]. All basic gene functions listed were found using GeneCards [33]. STRING included the most widespread enrichment analysis, so all significant STRING enrichments for gene groups of interest are reported in the additional information.

## Results

### Males and females overlap in their patterns of differential gene expression

A full list of genes from most to least differentially expressed with directionality information of expression change (upregulated in high-singers = positive sign, downregulated in high-singers = negative sign) is provided in Supplementary Material S2. In males, 51 genes had an adjusted p-value (Benjamini-Hochberg False Discovery Rate (FDR)) of less than 0.05, with 1,389 genes having a raw p-value of less than 0.05. In females, only 4 genes had an adjusted p-value of less than 0.05 and had 888 genes with a raw p-value less than 0.05.

**Fig. 3:**
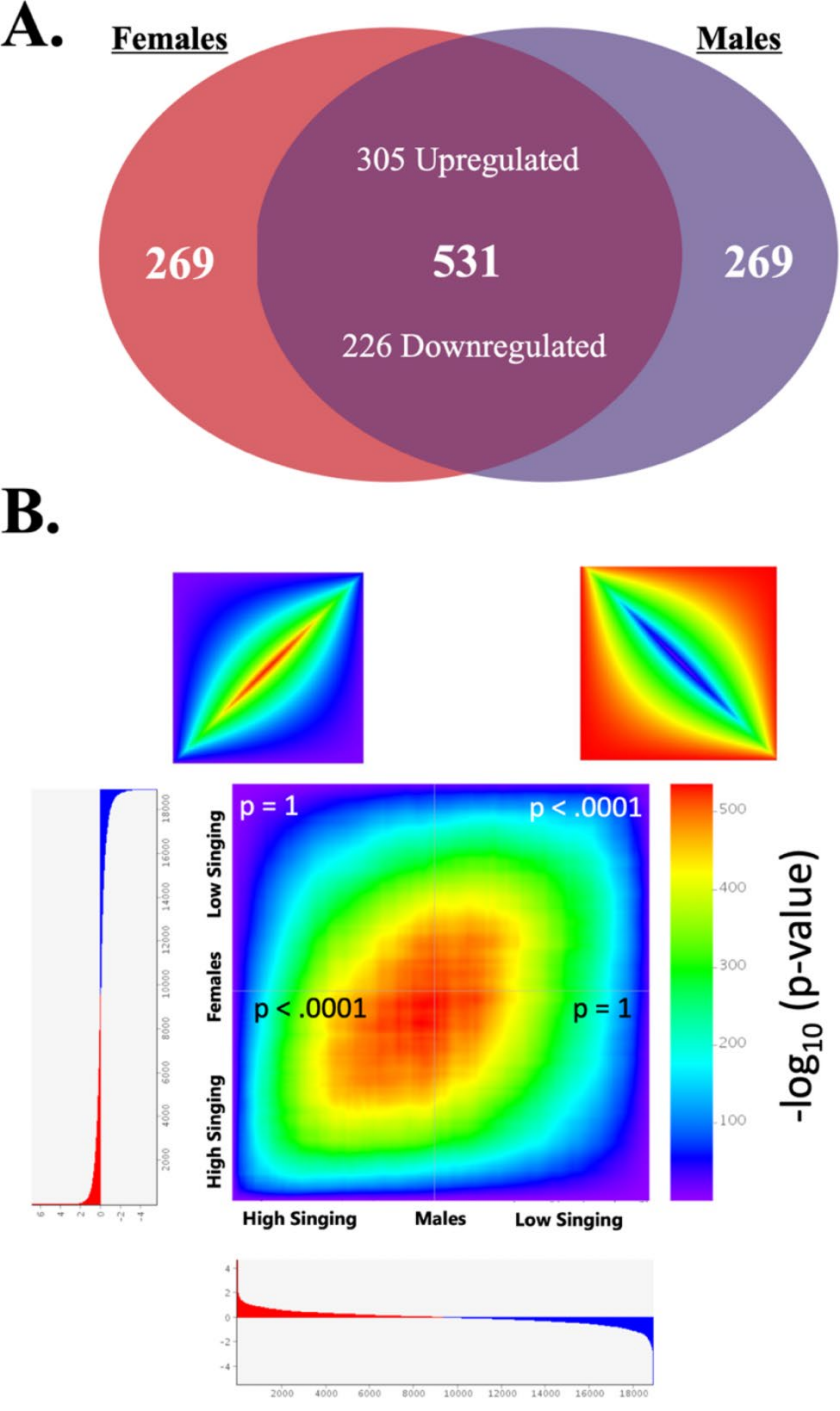
**(A)** Venn diagram showing the overlap in expression of the 800 most differentially expressed genes relative to high singers when comparing males and females. **B)** Heatmap generated by rank-rank hypergeometric overlap analysis comparing high-singing and low-singing males and females in the medial preoptic area (mPOA) with p-values indicating the significance of the overlap between the top 800 most differentially expressed genes within each quadrant. Warmer colors represent higher proportions of overlap, while cooler colors represent lower portions of overlap. Examples of perfect correlations and perfect anti-correlations are included above for reference. The mPOA shows very similar expression profiles in males and females, with significant overlap in concordant gene expression, but no significant overlap in discordant gene expression

Previous work has shown that top differentially expressed genes that did not meet threshold for FDR were successfully validated with qPCR [34], so we expect many genes with a raw p-value of 0.05 to still be biologically meaningful. Due to the large amount of differentially expressed genes with a p-value of less than 0.05, we performed rank-rank hypergeometric overlap analysis to uncover genes concordant in males and females. Males and females showed remarkable similarity to one another, with high-singing and low-singing groups showing significant overlap across sexes (low-singers: p < .0001, high-singers: p < .0001) (Fig. 3). Out of the top 800 most differentially expressed genes (~ top 7% of annotated genome), we found 305 genes consistently upregulated and 226 genes consistently down-regulated in male and female high-singing birds. Enrichment analysis in STRING for concordant expressing genes across sexes showed significant enrichment for neuromodulators associated with motivated behaviors such as dopamine and acetylcholine as well as enrichment for GABA and glutamate (Table 1). Additionally, ToppCluster-enrichment showed many genes associated with autism, wherein affiliative social communication is heavily impacted.

**Table 1:** Genes of interest that differ in the mPOA of low- and high-singing European starlings

Gene Symbol	Gene	Function	Group difference	p-value
**Acetylcholine-related**				
CHAT	choline O-acetyltransferase	Catalyzes acetylcholine production	High > Low	female = 0.009, male = 0.001
SLC5A7	solute carrier family 5 member 7	High-affinity choline transporter	High > Low	female = 0.002, male = 0.012
CHRM4	cholinergic receptor muscarinic 4	Acetylcholine receptor	High > Low	female = 0.026, male < 0.001*
**Dopamine-related**				
SLC18A2	Solute Carrier Family 18 Member A2	Monoamine transporter	Low > High	female = 0.004, male = 0.003
DRD2	Dopamine Receptor D2	Dopamine receptor	High > Low	female = 0.087, male = 0.036
DRD5	Dopamine Receptor D5	Dopamine receptor	High > Low	female = 0.03, male < 0.001
NTSR1	Neurotensin Receptor 1	Neurotensin receptor	High > Low	female = 0.07, male = 0.017
PPP3CC	Protein Phosphatase 3 Catalytic Subunit Gamma	Downstream regulation of dopaminergic signaling	High > Low	female = 0.045 male = 0.029
**GABA-related**				
GABRB3	Gamma-Aminobutyric Acid Type A Receptor Subunit Beta3	GABA receptor subunit	High > Low	female = 0.012, male = 0.004
GABRA5	Gamma-Aminobutyric Acid Type A Receptor Subunit Alpha5	GABA receptor subunit	High > Low	female = 0.066, male < 0.001*
GABRD	Gamma-Aminobutyric Acid Type A Receptor Subunit Delta	GABA receptor subunit	High > Low	female = 0.014, male = 0.004
LRRTM1	Leucine Rich Repeat Transmembrane Neuronal 1	Regulates presynapse assembly in GABA synapses	High > Low	female = 0.03, male = 0.029
**Glutamate-related**				
GRIN1	Glutamate Ionotropic Receptor NMDA Type Subunit 1	Glutamate receptor subunit	High > Low	female = 0.03, male = 0.002
GRIK3	Glutamate Ionotropic Receptor Kainate Type Subunit 3	Glutamate receptor subunit	High > Low	female = 0.047, male = 0.007
GRIA2	Glutamate Ionotropic Receptor AMPA Type Subunit 2	Glutamate receptor subunit	High > Low	female = 0.011, male < 0.001
GRM5	Glutamate Metabotropic Receptor 5	Glutamate receptor	High > Low	female = 0.051, male = 0.003
GRIA1	Glutamate Ionotropic Receptor AMPA Type Subunit 1	Glutamate receptor subunit	High > Low	female = 0.011, male < 0.001
SHANK2	SH3 And Multiple Ankyrin Repeat Domains 2	Encodes scaffolding for excitatory synapse	High > Low	female = 0.011, male = 0.004

*Indicates significant false discovery rate

Our DGE analysis comparing males and females found 1,194 genes with a raw p-value less than 0.05, with 685 of these genes significantly upregulated in males relative to females, and 509 upregulated in females relative to males. Thirty-eight of the genes significantly upregulated in females were linked to the W chromosome (ChrW) of the 2021 Zebra finch NCBI genome annotation. These genes did not contain any notable enrichment. In this annotation, there were 165 ChrW-linked genes, but 123 of these genes were excluded due to low counts across groups. This left 4 ChrW genes not significantly upregulated in females relative to males. Three hundred eighty-three of the genes upregulated in males were linked to the Z chromosome (ChrZ). These genes were enriched for multiple metabolic processes. There were 1084 ChrZ-linked genes for this annotation, but 508 did not have differential expression data due to low counts. This left 193 ChrZ genes not significantly upregulated in males relative to females. Full EdgeR results of DGE analysis comparing males and females (upregulated in females = positive sign, upregulated in males = negative sign) can also be found in Supplementary Material S2.

### Birds show differences in gene connectivity across singing condition

For the initial WGCNA analysis, 15 modules were identified (see Supplementary Material S3), however, no module eigengenes significantly correlated with song. Although it did not reach significance (p = .086), the greenyellow module was most closely associated with song and consisted of 93 genes. It contained three glutamatergic signaling genes (GRM2, GRIN2A, SLC17A6), a cholinergic receptor gene (CHRNA5), and a GABA receptor subunit (GABRD).

**Fig. 4:**
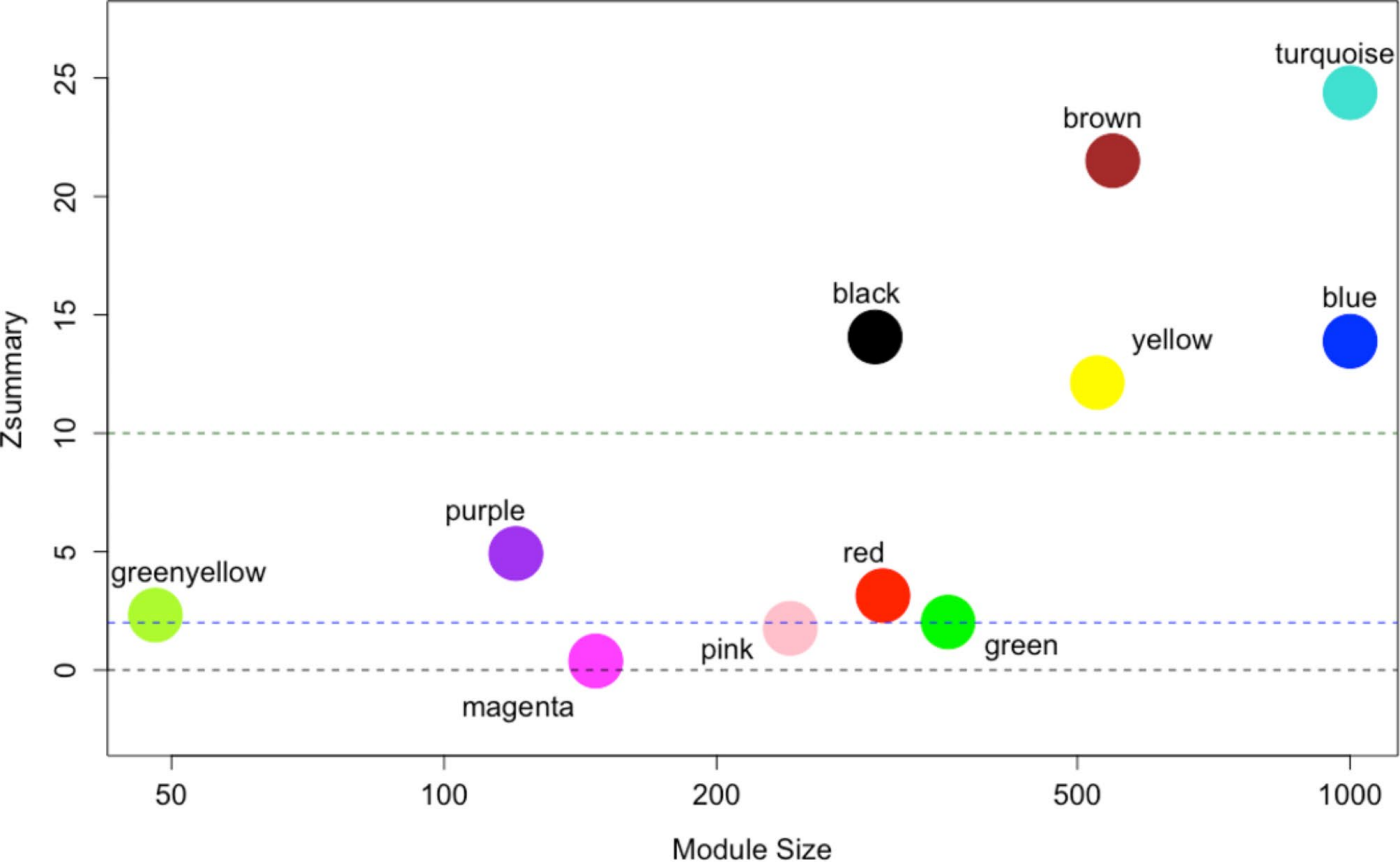
WGCNA module preservation analysis results between low-singing and high-singing starlings depicted as a Zsummary, a composite value of Z-statistics representing module connectivity and density, mapped against module size. The grey module is excluded. If a module had a Zsummary < 2, it was considered not preserved across conditions. Pink and magenta were the only modules not preserved across condition, while all other modules maintained some form of preservation in both singing groups

**Fig. 5:**
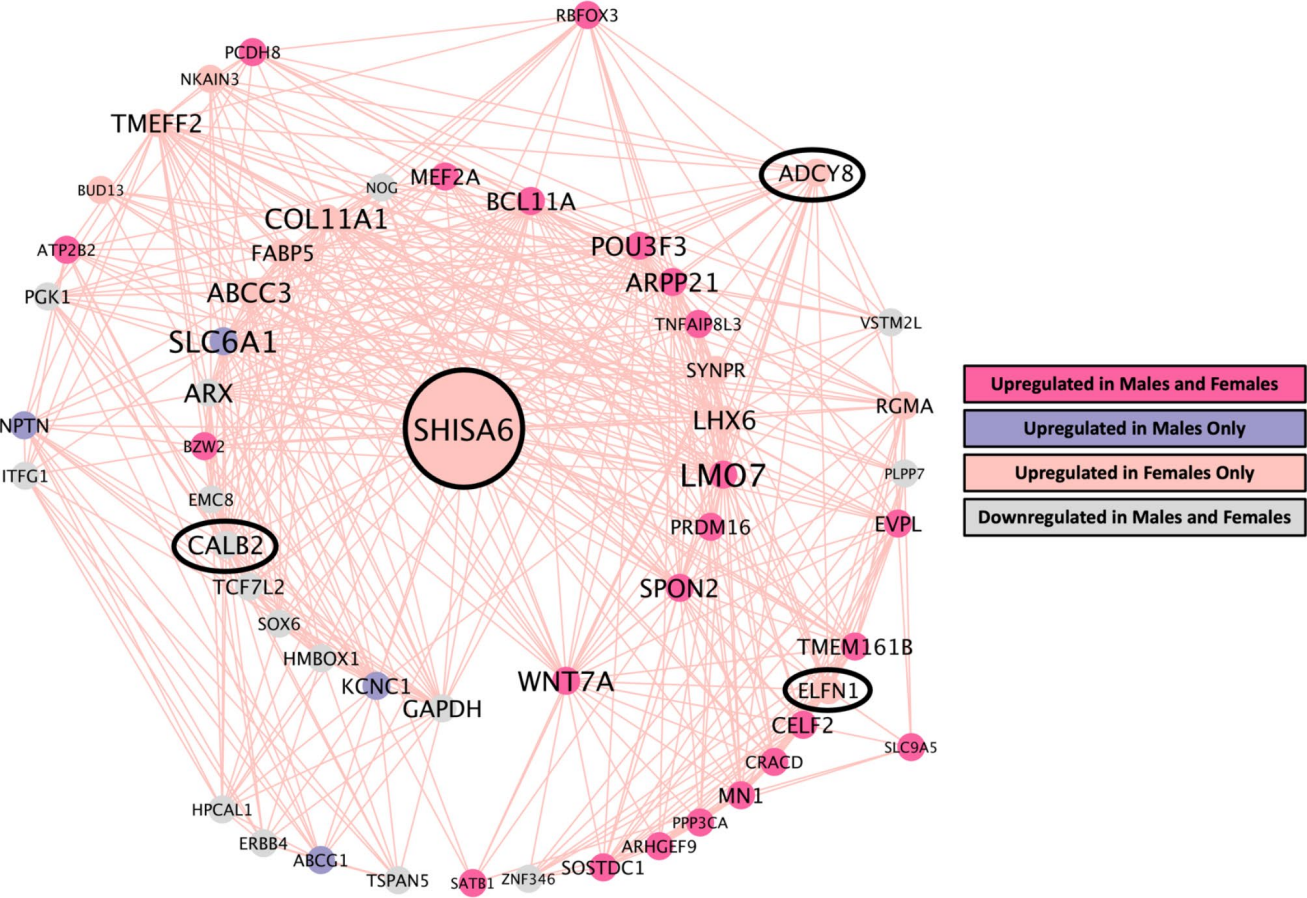
Top connected genes in the WGCNA pink module, a module that was not preserved across singing condition in the mPOA. Genes with less than 15 connections were excluded from visualization. Increased font size for the gene symbols indicates a greater number of connections between genes. Genes previously identified for a role in glutamatergic signaling are indicated by black circles. The large gene in the center (SHISA6) is the hub gene of this module identified by WGCNA. Protocols for identifying modules of interest are provided in the “Methods” section

For the module preservation analysis, we found multiple modules that were not preserved between low- and high- singers. For the high-singer dataset, WGCNA identified 16 modules of genes, while 12 modules were identified in the low-singer dataset. Module preservation analysis identified 3 gene modules with a Zsummary < 2 and were therefore not preserved between the two analyses (Fig. 4). All module preservation analysis results and genes in the modules of interest can be found in Supplementary Material S3. One of these 3 was the grey module, which consists of genes that are “unassigned” to any module produced, so this module was not investigated further. The non-preserved pink module consisted of 241 genes and was significantly enriched (in STRING) for glutamate-related genes (Fig. 5). This includes CACNG3 and CACNG5, both AMPA receptor regulatory proteins, and this module’s hub gene (the most connected gene) was SHISA6, which is thought to enable ionotropic glutamate receptor binding activity. The pink module also showed enrichment for GABAergic synapse, containing the GABA transporter SLC6A1 as well as the GABA receptor subunit GABRD, which was one of the previously identified differentially expressed genes. The pink module contained the immediate early gene EGR1, growth-hormone inhibiting hormone SST, in addition to two estrogen-related genes. This included GEPR1, an estrogen receptor, and CITED4, which enhances estrogen-dependent transactivation. The magenta module of 147 genes was also not preserved across singing condition, however, was not significantly enriched for any notable neural pathways.

### A co-expressing module of glutamatergic genes significantly correlated with song

MEGENA identified 542 nested modules. CTA identified 145 of these modules’ eigengenes (1st principal component) that significantly correlated with singing condition. Due to the numerous modules related to behavior, we performed an overlap analysis using the GeneOverlap package [35] to determine which of these modules were significantly enriched for the 305 differentially-expressed genes in high singers. In our efforts to minimize interpretational noise due to extremely large module size, we further filtered those modules by removing those that contained more than 150 genes and had less than 5 differentially expressed genes, leaving 30 modules of interest (see Supplementary Material S4).

**Fig. 6:**
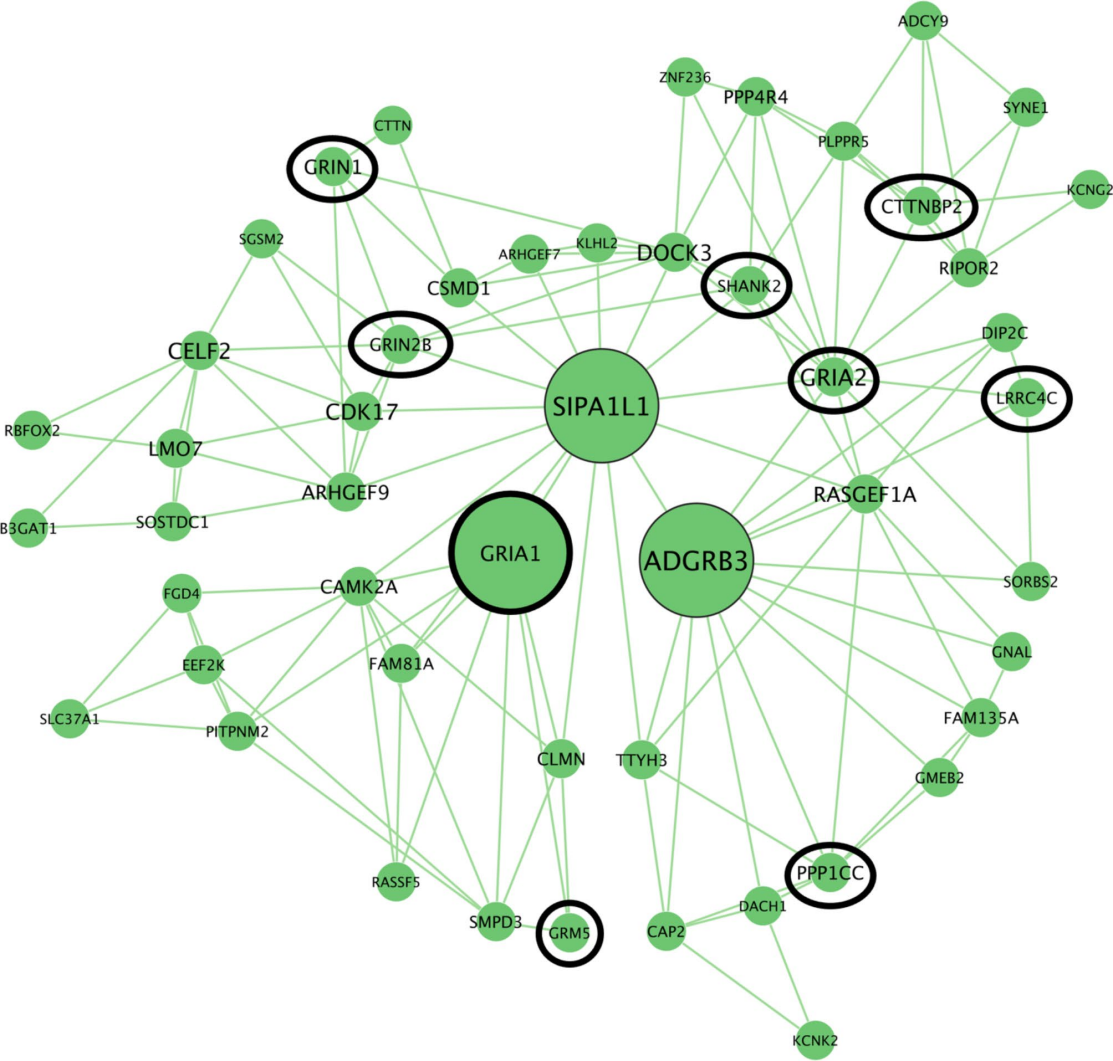
Top connected genes in MEGENA module #237, whose eigengene correlated with singing condition in the mPOA. All genes were upregulated in both males and females. Increased font size for the gene symbols indicates a greater number of connections between genes. Genes with fewer than 5 connections were excluded from visualization. Genes previously identified for a role in glutamatergic signaling are indicated by black circles. The large genes in the center (GRIA1, SIPA1L1, ADGRB3) are the hub genes of this module identified by MEGENA. Protocols for identifying modules of interest are provided in the “Methods” section

**Fig. 7:**
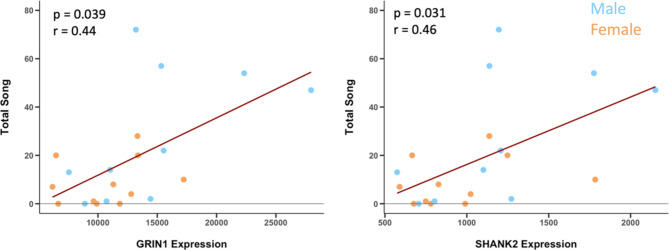
Pearson correlations between the expression (RSEM expected counts) of two glutamate related genes (GRIN1, SHANK2) and total song. Both genes were part of MEGENA modules whose eigengene significantly correlated with singing condition in cluster trait analysis. Total song is total number of point samples recorded over 5 days of observation. Dark red line indicates significant correlation based on raw p-value. Please note that these do not include corrections for multiple testing

Seven of these modules were significantly enriched in STRING for glutamate pathways. Module #237 contained multiple glutamate receptor genes (GRM5, GRIA1, GRIA2, GRIN1, GRIN2B) and glutamate synapse scaffolding genes (SHANK2, HOMER2). This module contained genes that were significantly enriched for genetic pathways previously identified in human neurodevelopmental disorders linked to social impairments. Hub genes for module #237 were GRIA1, ADGRB3, and SIPA1L1 (Fig. 6). Two other modules were significantly enriched for thyroid hormone receptor binding including nuclear receptor co-repressor and co-activator (NCOR1, NCOA6). See Table 2 for information regarding the parent module (the larger module that a module of interest is nested in), hub genes, and eigengene-song p-values for standout MEGENA modules of interest. We plotted the relationships between the genes of interest identified by MEGENA and found significant positive Pearson correlations between song and GRIN1 and SHANK2 expression (Fig. 7) based on the raw p-value, however, we did not correct for multiple testing. The other 21 modules showed no notable significant enrichment and were not investigated further.

**Table 2:** MEGENA modules of interest

Module	Parent Module	Number of Genes in Module	Hub Genes (number of connections in module)	Eigengene-Song p-value
31	8	59	KCNS2(17) HIVEP2(14) FLRT2(14)	0.027
203	31	54	KCNS2(17)	0.026
237	48	114	ADGRB3(22) SIPA1L1(21) GRIA2(17)	0.025
602	237	16	GRIA1(12)	0.021
603	237	17	DOCK3(11)	0.01
300	71	17	EPHA7(10)	0.011
345	84	115	NCOA6(21) CELSR3(20) RBM5(18) RAP2A(17) KMT2A(16)	0.043
783	345	99	NCOA6(21) CELSR3(20) RBM5(18) RAP2A(17) KMT2A(16)	0.027
572	203	46	KCNS2(17) HIVEP2(14) FLRT2(14)	0.028

## Discussion

Using multiple bioinformatics approaches we have identified several candidate genes associated with the production of gregarious song in European starlings. Male and female birds show consistent patterns of song-associated gene expression, with elevated dopamine-, acetylcholine-, and GABA-related expression in the mPOA associated with high singing rates. We also identified a co-expressing complex of glutamate-related genes closely associated with song, most of which have been previously linked to phenotypes wherein social interactions are heavily dysregulated [36, 37].

### Male and female singing groups show remarkably similar gene expression profiles

Our comparison of DGE results through RRHO revealed that low- and high-singing birds had similar genes upregulated and downregulated across sexes. We found this particularly interesting as low- and high-singing groups were created by median split of singing rate within each sex, not a median split across singing rates for all birds combined. In terms of raw singing rate, high-singing females (mean song = 17.2) were less similar to high-singing males (mean = 64.8) than low-singing males (mean = 8.667). Despite this, our RRHO comparison showed that the high-singing females’ expression profile was more similar to that of high-singing males, with over 500 genes in concordant expression out of the top 750 most differentially expressed genes. This is noteworthy as it suggests that these genes are not simply representative of motor activity.

We did not find any obvious sex differences in song-related directionality as our most differentially expressed genes across singing condition remained largely the same across males and females. However, in our DGE analysis comparing males and females independent of singing condition, we found over 200 genes significantly differentially expressed between sexes. Although many of these genes were enriched for various metabolic processes, we did not find significant differences in sex steroid specific metabolism genes, such as CYP19A1 or the SRD5A variants (see Supplementary Material files), which encode for aromatase and 5a-reductase respectively. This may be surprising given that the mPOA is a steroid sensitive, sexually differentiated brain region [38, 39]. However, sex differences in protein or gene expression in the mPOA are commonly linked to steroid hormone concentrations [40], which are extremely low in non-breeding condition starlings [41]. It thus may be in the absence of high circulating testosterone and estradiol that sexually differentiated expression related to sex steroids is absent. We do see that androgen receptor (AR) and estrogen receptor (ESR1 and ESR2) expression may be marginally higher in female high-singers, but not in males. The biological relevance of these differences must be explored in future research [10, 42].

We also found that 38 genes linked to the avian female-specific ChrW were upregulated in females relative to males. This result is consistent with the expectations set by the sex-specific nature of the ChrW [43, 44]. Only 4 ChrW genes analyzed by DGE did not show significant differential expression across the sexes and 123 had insufficient counts to perform DGE analysis. Considering there is limited female-specific positive selection of genes on the avian ChrW, this outcome is not unexpected [45]. It is plausible that many of these genes may have redundant paralogs on the Z chromosome and others, reducing detectable expression differences across sexes. These findings contribute to a growing body of knowledge regarding the female songbirds’ genome that has been historically understudied.

### Neurotransmitter systems previously implicated in social behavior are differentially expressed across singing condition

We found that multiple dopamine-related genes were differentially expressed across singing condition. These included D5 (a D1-like receptor) and D2 receptors, a dopamine transporter (SLC18A2), a protein phosphatase (PPP3CC), and neurotensin receptor (NTSR1). Dopamine in the mPOA in birds and rodents stimulates sexual motivation [46–48], including sexually-motivated birdsong [49]; and the mPOA directly accesses the mesolimbic dopaminergic system through projections to the ventral tegmental area (VTA), which is well-known to be involved in motivation in numerous sexual and non-sexual contexts [50–52]. It is thus possible that differential patterns of dopamine-related gene expression in the mPOA regulates motivational aspects of singing behavior.

Prior research on starlings demonstrates that optimal levels of D1 dopamine receptor stimulation in the mPOA facilitate sexually-motivated male song [53, 54]; however, its role in gregarious song has not been extensively studied. Previous research has found strong, positive linear correlations between D1- but not D2-like receptor density within the mPOA and gregarious song in male starlings [11]. This is inconsistent with recent work studying undirected song in zebra finches, which is similar to gregarious song in that it is performed in flocks in a non-sexual context [5, 55]. It was found that peripheral administration of the D2 receptor antagonist haloperidol reduced the rate of undirected song in male zebra finches, while the D1 receptor antagonist SCH23390 had no effect [56]. One likely reason of these conflicting findings is simply because antagonists were not injected directly within the mPOA; however, the effects of dopamine receptor manipulations in mPOA on gregarious song have not been tested. Our findings that D5 and D2 are upregulated in birds singing high rates of gregarious song suggest that dopamine receptors in mPOA may regulate song across contexts but this must be experimentally tested.

We also found multiple acetylcholine genes upregulated in high-singing starlings, including choline O-acetyltransferase (CHAT), a choline transporter (SLC5A7), and a muscarinic cholinergic receptor (CHRM4). Multiple immunolabeling studies have shown high concentrations of cholinergic proteins in the song control system in the zebra finch brain [57–59], and cholinergic stimulation of the HVC via carbachol modulates zebra finch song [60]. However, the literature regarding its role in brain regions involved in social behavior is limited. A study in rats found that a direct injection of a cholinergic receptor agonist into the anterior hypothalamic-preoptic area induced 22 kHz ultrasonic vocalizations, which are associated with an anxiety-like, negative emotional state [61, 62]. Although gregarious song is a vocalization associated with positive emotional state [5], it may be that acetylcholine acts within the mPOA to induce emotionally-salient vocal behavior, however, this needs to be investigated further.

Genes for GABA Type A (GABA_A_) receptor subunit beta3 (GABRB3), alpha5 (GABRA5), and delta (GABRD) were upregulated in high-singing birds. GABRD was also a member of the module most associated with song in our first WGCNA analysis. Individual gene deficiencies in both GABRA5 and GABRB3 are strongly associated with reduced prosocial behavior in mice, including reductions in social exploration, contact, and vocalizations [63–65]. Similarly, dysregulated GABRD expression throughout pregnancy causes mouse dams to keep an increased distance from their pups postpartum [66]. These studies involved global manipulation of these genes and therefore did not identify specific regions of action. Our findings suggest that the mPOA is one region wherein GABA_A_ receptor binding may influence social interactions, making it a promising region to perform viral manipulations of GABA_A_ subunit receptor genes to understand their specific contributions to the regulation of behavior across social contexts.

Given that a body of research, reviewed in the introduction, demonstrates a role for mu opioid receptors in the mPOA in gregarious song and the reward that accompanies singing behavior, it was somewhat surprising that opioid-specific genes did not turn up in our analyses. This was unexpected, as downregulation of mu opioid receptors in the mPOA reduced song [8]. However, a previous study in the nucleus accumbens revealed the effect of opioid receptor stimulation on gregarious song to be small, only occurring when the highest receptor agonist dose was given [15]. Given this, it is possible that the expression signal of opioid related genes in the mPOA may also not have been a high enough magnitude to be detected by RNA-seq. Additionally, past studies reveal curvilinear, inverted U-shaped relationships between mu opioid receptors and gregarious song [67], which may be missed by the current analysis and is something to examine in future studies.

### Multiple approaches implicate glutamatergic signaling genes in the mPOA in the production of gregarious song

DGE, RRHO, and both co-expression network analyses we performed uncovered numerous glutamate-related genes related to singing condition. This is particularly of note, as WGCNA and MEGENA use single-scale and multi-scale topologies, respectively, to cluster functionally related genes together, and both analyses highlighted glutamatergic systems as being related to singing condition. Many of these glutamatergic-related genes coincided with enriched pathways for major depressive disorder and neurodevelopmental disorders, which are both characterized by social dysregulation, and depression is specifically associated with anhedonia [68–70]. Given that gregarious song is highly social and tightly associated with a reward (i.e., hedonic) state [10, 71], these findings suggest that the mPOA is a key, yet understudied, site in which glutamate regulation acts to influence rewarding, gregarious social interactions [5].

One of the modules related to song that we identified using MEGENA contained seven glutamate receptor and synapse scaffolding genes, all of which have been genes of interest in studies of neurodevelopmental disorders with socio-communicative deficits [36, 37, 72, 73]. One gene functionally implicated in the regulation of social interactions we found that significantly correlated with song was NMDA receptor subunit, GRIN1. GRIN1 knockdown mice have been identified and used as an autism model [37, 74, 75], and loss of GRIN1 activation in corticotropin-releasing factor neurons in male naïve mouse increases social stress [76]. Another functionally similar gene we found correlated with gregarious song was SHANK2, a glutamatergic synapse scaffolding gene [77, 78]. SHANK2 knockdown mice have also been proposed as an autism model [36, 79, 80], and its knockdown has been shown to induce deficits in sociability in naïve mice [81] and reduce parental bond in dams [82]. Interestingly, it was found that activation of the mPOA via DREADDS reestablishes social bonding in these mothers, highlighting the region-specific importance of SHANK2 in affiliative interactions. Our identification of GRIN1 and SHANK2 as possible candidate genes that act within the mPOA to regulate gregarious behaviors is consistent with this literature.

One hub gene identified in WGCNA associated with singing condition was SHISA6, an auxiliary AMPA receptor subunit [83], while another hub gene identified in MEGENA was GRIA1, another AMPA receptor subunit. Although there is no research on the functional role of SHISA6 in the mPOA, recent work in mice has shown that increased SHISA6 expression in the nucleus accumbens (NAc) is linked to decreased social interaction in mice [84]. Additionally, antagonism of AMPA receptors in the NAc increases social interaction in social avoidant rats [85]. The NAc is indirectly connected to the mPOA, as it is part of a motivation and reward circuit that receives input from the VTA, which as previously mentioned, receives input from the mPOA [50, 51, 86]. The NAc also contains direct, reciprocal connections with both the VTA and mPOA [87–89]. The NAc has also been shown to regulate gregarious song in starlings [10, 15, 90], so our mPOA results suggest the possibility that AMPA receptors act across this reward circuit to modulate the production of song. Due to their identification as hub genes associated with the glutamatergic AMPA receptor complex, SHISA6 and GRIA1 standout as excellent candidates for direct manipulation in the mPOA to uncover their specific role in the motivation and production of gregarious song.

## Conclusions

Using multiple bioinformatics approaches, we have identified multiple candidate genes within the mPOA that may play a role in producing gregarious song in starlings further enhancing our understanding of the genetic underpinnings of a complex social behavior. Standout genes include DRD2, GRIN1, and SHANK2, due to their previously identified in role in prosocial, affiliative behaviors. Although RNA-seq allows for a thorough analysis of the neuromodulators within a given region, it is important to note that this research is exploratory in nature and targeted manipulations of these candidate genes are required before drawing definitive conclusions of their role in gregarious song. Additionally, this study used bulk RNA-seq, which does not consider the various cell types in the mPOA. In an analysis of the mouse POA, over 70 different neuron and glial cell types were identified [91]. The starling mPOA likely contains similar cellular diversity, so it is possible our analysis missed song-related genes that are limited to specific cell-types. Lastly, we did not perform individual hub gene validation using common methods such as quantitative polymerase chain reaction (qPCR). We note that the effectiveness of qPCR to validate RNA-seq is not clear due to differences in probe bias between the methods [92–95], so we do not believe this reduces the validity of our findings.

This study provides a detailed characterization of the genetic profile of an integral part of the brain’s social behavior network, and the included data will allow other researchers to examine and compare genes across other social behaviors and species. Furthermore, because of the highly conserved nature of the mPOA across vertebrates, our candidate genes of interest may regulate prosocial, affiliative behaviors across taxa.

### Electronic supplementary material

Below is the link to the electronic supplementary material.

Supplementary Material 1: RSEM expected count values and behavioral data.

Supplementary Material 2: All differential gene expression results.

Supplementary Material 3: All WGCNA results.

Supplementary Material 4: All MEGENA results.

## Data Availability

All datasets generated and/or analyzed during the current study are included in this published article [and its supplementary information files].

## References

[1] Bradbury JW, Vehrencamp SL. Principles of animal communication. 1998.

[2] Vanderschuren LJ, Trezza V (2014). What the laboratory rat has taught us about social play behavior: role in behavioral development and neural mechanisms. Curr Top Behav Neurosci.

[3] Himmler BT, Pellis SM, Kolb B (2013). Juvenile play experience primes neurons in the medial prefrontal cortex to be more responsive to later experiences. Neurosci Lett.

[4] Hausberger M, Richard-Yris M-A, Henry L, Lepage L, Schmidt I (1995). Song sharing reflects the social organization in a captive group of european starlings (Sturnus vulgaris). J Comp Psychol.

[5] Riters LV, Polzin BJ, Maksimoski AN, Stevenson SA, Alger SJ. Birdsong and the neural regulation of positive emotion. Front Psychol 2022, 13. 10.3389/fpsyg.2022.903857 PMC9258629 35814050

[6] Alger SJ, Riters LV (2006). Lesions to the medial preoptic nucleus differentially affect singing and nest box-directed behaviors within and outside of the breeding season in european starlings (Sturnus vulgaris). Behav Neurosci.

[7] Polzin BJ, Maksimoski AN, Stevenson SA, Zhao C, Riters LV. Mu opioid receptor stimulation in the medial preoptic area or nucleus accumbens facilitates song and reward in flocking european starlings. Front Physiol:1912. 10.3389/fphys.2022.970920 PMC9510710 36171974

[8] Stevenson SA, Piepenburg A, Spool JA, Angyal CS, Hahn AH, Zhao C, Riters LV (2020). Endogenous opioids facilitate intrinsically-rewarded birdsong. Sci Rep.

[9] Riters LV, Stevenson SA, Devries MS, Cordes MA (2014). Reward Associated with singing behavior correlates with opioid-related gene expression in the Medial Preoptic Nucleus in male european starlings. PLoS ONE.

[10] Polzin BJ, Maksimoski AN, Stevenson SA, Zhao C, Riters LV. Mu opioid receptor stimulation in the medial preoptic area or nucleus accumbens facilitates song and reward in flocking european starlings. Front Physiol 2022, 13. 10.3389/fphys.2022.970920 PMC9510710 36171974

[11] Heimovics SA, Cornil CA, Ball GF, Riters LV (2009). D1-like dopamine receptor density in nuclei involved in social behavior correlates with song in a context-dependent fashion in male european starlings. Neuroscience.

[12] Hahn AH, Merullo DP, Spool JA, Angyal CS, Stevenson SA, Riters LV (2017). Song-associated reward correlates with endocannabinoid-related gene expression in male european starlings (Sturnus vulgaris). Neuroscience.

[13] Heimovics SA, Cornil CA, Ellis JMS, Ball GF, Riters LV (2011). Seasonal and individual variation in singing behavior correlates with alpha 2-noradrenergic receptor density in brain regions implicated in song, sexual, and social behavior. Neuroscience.

[14] Goodson JL (2005). The vertebrate social behavior network: evolutionary themes and variations. Horm Behav.

[15] Maksimoski AN, Polzin BJ, Stevenson SA, Zhao C, Riters LV. µ-Opioid Receptor Stimulation in the Nucleus Accumbens Increases Vocal–Social Interactions in Flocking European Starlings, Sturnus Vulgaris. *eneuro* 2021, 8(5):ENEURO.0219–0221. 10.1523/ENEURO.0219-21.2021 PMC8474649 34475266

[16] Zhao C, Chang L, Auger AP, Gammie SC, Riters LV. Mu opioid receptors in the medial preoptic area govern social play behavior in adolescent male rats. Genes Brain and Behavior 2020, 19(7). 10.1111/gbb.12662 PMC7643862 32388931

[17] Dawson A, King VM, Bentley GE, Ball GF (2001). Photoperiodic Control of Seasonality in Birds. J Biol Rhythm.

[18] Kovács KJ (1998). Invited review c-Fos as a transcription factor: a stressful (re) view from a functional map. Neurochem Int.

[19] Zhao C, Saul MC, Driessen T, Gammie SC (2012). Gene expression changes in the septum: possible implications for microRNAs in sculpting the maternal brain. PLoS ONE.

[20] Zhao C, Chang L, Auger AP, Gammie SC, Riters LV (2020). Mu opioid receptors in the medial preoptic area govern social play behavior in adolescent male rats. Genes Brain and Behavior.

[21] Li B, Dewey CN (2011). RSEM: accurate transcript quantification from RNA-Seq data with or without a reference genome. BMC Bioinformatics.

[22] Robinson MD, McCarthy DJ, Smyth GK (2010). edgeR: a Bioconductor package for differential expression analysis of digital gene expression data. Bioinformatics.

[23] Plaisier SB, Taschereau R, Wong JA, Graeber TG (2010). Rank–rank hypergeometric overlap: identification of statistically significant overlap between gene-expression signatures. Nucleic Acids Res.

[24] Langfelder P, Horvath S (2008). WGCNA: an R package for weighted correlation network analysis. BMC Bioinformatics.

[25] WGCNA Package FAQ. [https://horvath.genetics.ucla.edu/html/CoexpressionNetwork/Rpackages/WGCNA/faq.html].

[26] Langfelder P, Luo R, Oldham MC, Horvath S (2011). Is my Network Module Preserved and Reproducible?. PLoS Comput Biol.

[27] Song W-M, Zhang B (2015). Multiscale embedded gene co-expression network analysis. PLoS Comput Biol.

[28] McKenzie AT, Katsyv I, Song W-M, Wang M, Zhang B (2016). DGCA: a comprehensive R package for differential gene correlation analysis. BMC Syst Biol.

[29] Kaimal V, Bardes EE, Tabar SC, Jegga AG, Aronow BJ (2010). ToppCluster: a multiple gene list feature analyzer for comparative enrichment clustering and network-based dissection of biological systems. Nucleic Acids Res.

[30] Ashburner M, Ball CA, Blake JA, Botstein D, Butler H, Cherry JM, Davis AP, Dolinski K, Dwight SS, Eppig JT (2000). Gene ontology: tool for the unification of biology. Nat Genet.

[31] The Gene Ontology (2021). Resource: enriching a GOld mine. Nucleic Acids Res.

[32] Szklarczyk D, Gable AL, Lyon D, Junge A, Wyder S, Huerta-Cepas J, Simonovic M, Doncheva NT, Morris JH, Bork P (2019). STRING v11: protein–protein association networks with increased coverage, supporting functional discovery in genome-wide experimental datasets. Nucleic Acids Res.

[33] Safran M, Dalah I, Alexander J, Rosen N, Iny Stein T, Shmoish M, Nativ N, Bahir I, Doniger T, Krug H. GeneCards Version 3: the human gene integrator. *Database* 2010, 2010. 10.1093/database/baq020 PMC2938269 20689021

[34] Zhao C, Eisinger BE, Driessen TM, Gammie SC. Addiction and reward-related genes show altered expression in the postpartum nucleus accumbens. Front Behav Neurosci 2014, 8. 10.3389/fnbeh.2014.00388 PMC4220701 25414651

[35] Shen L. GeneOverlap: an R package to test and visualize gene overlaps. R Package 2014, 3.

[36] Won H, Lee H-R, Gee HY, Mah W, Kim J-I, Lee J, Ha S, Chung C, Jung ES, Cho YS (2012). Autistic-like social behaviour in Shank2-mutant mice improved by restoring NMDA receptor function. Nature.

[37] Chung W, Choi SY, Lee E, Park H, Kang J, Park H, Choi Y, Lee D, Park S-G, Kim R (2015). Social deficits in IRSp53 mutant mice improved by NMDAR and mGluR5 suppression. Nat Neurosci.

[38] Gorski RA, Gordon JH, Shryne JE, Southam AM (1978). Evidence for a morphological sex difference within the medial preoptic area of the rat brain. Brain Res.

[39] Arendash GW, Gorski RA (1983). Effects of discrete lesions of the sexually dimorphic nucleus of the preoptic area or other medial preoptic regions on the sexual behavior of male rats. Brain Res Bull.

[40] Panzica G, Pessatti M, Viglietti-Panzica C, Grossmann R, Balthazart J (1999). Effects of testosterone on sexually dimorphic parvocellular neurons expressing vasotocin mRNA in the male quail brain. Brain Res.

[41] Dawson A, Goldsmith A (1983). Plasma prolactin and gonadotrophins during gonadal development and the onset of photorefractoriness in male and female starlings (Sturnus vulgaris) on artificial photoperiods. J Endocrinol.

[42] Cordes M, Stevenson S, Driessen T, Eisinger B, Riters L (2015). Sexually-motivated song is predicted by androgen-and opioid-related gene expression in the medial preoptic nucleus of male european starlings (Sturnus vulgaris). Behav Brain Res.

[43] Xu L, Auer G, Peona V, Suh A, Deng Y, Feng S, Zhang G, Blom MP, Christidis L, Prost S (2019). Dynamic evolutionary history and gene content of sex chromosomes across diverse songbirds. Nat Ecol Evol.

[44] Xu L, Irestedt M, Zhou Q (2020). Sequence transpositions restore genes on the highly degenerated W chromosomes of songbirds. Genes.

[45] Xu L, Zhou Q (2020). The female-specific W chromosomes of birds have conserved gene contents but are not feminized. Genes.

[46] Moses J, Loucks JA, Watson HL, Matuszewich L, Hull EM (1995). Dopaminergic drugs in the medial preoptic area and nucleus accumbens: effects on motor activity, sexual motivation, and sexual performance. Pharmacol Biochem Behav.

[47] Ball GF, Balthazart J (2004). Hormonal regulation of brain circuits mediating male sexual behavior in birds. Physiol Behav.

[48] Kleitz-Nelson HK, Dominguez JM, Ball GF (2010). Dopamine release in the medial preoptic area is related to hormonal action and sexual motivation. Behav Neurosci.

[49] Heimovics SA, Riters LV (2008). Evidence that dopamine within motivation and song control brain regions regulates birdsong context-dependently. Physiol Behav.

[50] McHenry JA, Otis JM, Rossi MA, Robinson JE, Kosyk O, Miller NW, McElligott ZA, Budygin EA, Rubinow DR, Stuber GD (2017). Hormonal gain control of a medial preoptic area social reward circuit. Nat Neurosci.

[51] Tobiansky DJ, Will RG, Lominac KD, Turner JM, Hattori T, Krishnan K, Martz JR, Nutsch VL, Dominguez JM (2016). Estradiol in the Preoptic Area regulates the dopaminergic response to Cocaine in the Nucleus Accumbens. Neuropsychopharmacology.

[52] Iyilikci O, Balthazart J, Ball GF. Medial preoptic regulation of the ventral tegmental area related to the control of sociosexual behaviors. Eneuro 2016, 3(6). 10.1523/ENEURO.0283-16.2016 PMC5220225 28083561

[53] Riters LV, Pawlisch BA, Kelm-Nelson CA, Stevenson SA (2014). Inverted-U shaped effects of D1 dopamine receptor stimulation in the medial preoptic nucleus on sexually motivated song in male european starlings. Eur J Neurosci.

[54] Devries MS, Cordes MA, Stevenson SA, Riters LV (2015). Differential relationships between D1 and D2 dopamine receptor expression in the medial preoptic nucleus and sexually-motivated song in male european starlings (Sturnus vulgaris). Neuroscience.

[55] Dunn AM, Zann RA (2010). Undirected song in wild Zebra Finch Flocks: Contexts and Effects of mate removal. Ethology.

[56] Kim Y, Kwon S, Rajan R, Mori C, Kojima S. Intrinsic motivation for singing in songbirds is enhanced by temporary singing suppression and regulated by dopamine. Sci Rep 2021, 11(1). 10.1038/s41598-021-99456-w PMC8514548 34645903

[57] Asogwa NC, Toji N, He Z, Shao C, Shibata Y, Tatsumoto S, Ishikawa H, Go Y, Wada K (2022). Nicotinic acetylcholine receptors in a songbird brain. J Comp Neurol.

[58] Ryan SM, Arnold AP (1981). Evidence for cholinergic participation in the control of bird song: acetylcholinesterase distribution and muscarinic receptor autoradiography in the zebra finch brain. J Comp Neurol.

[59] Watson JT, Adkins-Regan E, Whiting P, Lindstrom JM, Podleski TR (1988). Autoradiographic localization of nicotinic acetylcholine receptors in the brain of the zebra finch (Poephila guttata). J Comp Neurol.

[60] Jaffe PI, Brainard MS. Acetylcholine acts on songbird premotor circuitry to invigorate vocal output. eLife 2020, 9. 10.7554/eLife.53288 PMC7237207 32425158

[61] Brudzynski SM (1994). Ultrasonic vocalization induced by intracerebral carbachol in rats: localization and a dose-response study. Behav Brain Res.

[62] Brudzynski SM (2007). Ultrasonic calls of rats as indicator variables of negative or positive states: acetylcholine–dopamine interaction and acoustic coding. Behav Brain Res.

[63] Zurek AA, Kemp SW, Aga Z, Walker S, Milenkovic M, Ramsey AJ, Sibille E, Scherer SW (2016). Orser BA: α5GABAA receptor deficiency causes autism-like behaviors. Ann Clin Transl Neurol.

[64] DeLorey TM (2005). GABRB3 gene deficient mice: a potential model of autism spectrum disorder. Int Rev Neurobiol.

[65] Delorey T, Sahbaie P, Hashemi E, Homanics G, Clark J (2008). Gabrb3 gene deficient mice exhibit impaired social and exploratory behaviors, deficits in non-selective attention and hypoplasia of cerebellar vermal lobules: a potential model of autism spectrum disorder. Behav Brain Res.

[66] Maguire J, Mody I (2008). GABAAR plasticity during pregnancy: relevance to Postpartum Depression. Neuron.

[67] Kelm-Nelson CA, Stevenson SA, Cordes MA, Riters LV (2013). Modulation of male song by naloxone in the medial preoptic nucleus. Behav Neurosci.

[68] Noens IL, van Berckelaer-Onnes IA (2005). Captured by details: sense-making, language and communication in autism. J Commun Disord.

[69] Pearlman-Avnion S, Eviatar Z (2002). Narrative analysis in developmental social and linguistic pathologies: dissociation between emotional and informational language use. Brain Cogn.

[70] Kupferberg A, Bicks L, Hasler G (2016). Social functioning in major depressive disorder. Neurosci Biobehavioral Reviews.

[71] Riters LV, Stevenson SA (2012). Reward and vocal production: song-associated place preference in songbirds. Physiol Behav.

[72] Zoicas I, Kornhuber J (2019). The role of metabotropic glutamate receptors in Social Behavior in rodents. Int J Mol Sci.

[73] Lee K, Goodman L, Fourie C, Schenk S, Leitch B, Montgomery JM (2016). AMPA receptors as therapeutic targets for neurological disorders. Adv protein Chem Struct biology.

[74] Teng BL, Nikolova VD, Riddick NV, Agster KL, Crowley JJ, Baker LK, Koller BH, Pedersen CA, Jarstfer MB, Moy SS (2016). Reversal of social deficits by subchronic oxytocin in two autism mouse models. Neuropharmacology.

[75] Burnashev N, Szepetowski P (2015). NMDA receptor subunit mutations in neurodevelopmental disorders. Curr Opin Pharmacol.

[76] Gilman TL, DaMert JP, Meduri JD, Jasnow AM (2015). Grin1 deletion in CRF neurons sex-dependently enhances fear, sociability, and social stress responsivity. Psychoneuroendocrinology.

[77] Sheng M, Kim E (2011). The postsynaptic Organization of Synapses. Cold Spring Harb Perspect Biol.

[78] Sheng M, Hoogenraad CC (2007). The postsynaptic architecture of excitatory synapses: a more quantitative view. Annu Rev Biochem.

[79] Lee Y-S, Yu N-K, Chun J, Yang J-E, Lim C-S, Kim H, Park G, Lee J-A, Lee K, Kaang B-K et al. Identification of a novel Shank2 transcriptional variant in Shank2 knockout mouse model of autism spectrum disorder. Mol Brain 2020, 13(1). 10.1186/s13041-020-00595-4 PMC7132969 32252796

[80] Arroyo-Araujo M, Graf R, Maco M, Van Dam E, Schenker E, Drinkenburg W, Koopmans B, De Boer SF, Cullum-Doyle M, Noldus LPJJ et al. Reproducibility via coordinated standardization: a multi-center study in a Shank2 genetic rat model for Autism Spectrum Disorders. Sci Rep 2019, 9(1). 10.1038/s41598-019-47981-0 PMC6690904 31406134

[81] Han KA, Yoon TH, Shin J, Um JW, Ko J. Differentially altered social dominance- and cooperative-like behaviors in Shank2- and Shank3-mutant mice. Mol Autism 2020, 11(1). 10.1186/s13229-020-00392-9 PMC7602353 33126897

[82] Grabrucker S, Pagano J, Schweizer J, Urrutia-Ruiz C, Schön M, Thome K, Ehret G, Grabrucker AM, Zhang R, Hengerer B et al. Activation of the medial preoptic area (MPOA) ameliorates loss of maternal behavior in a Shank2 mouse model for autism. EMBO J 2021, 40(5). 10.15252/embj.2019104267 PMC7917557 33491217

[83] Klaassen RV, Stroeder J, Coussen F, Hafner A-S, Petersen JD, Renancio C, Schmitz LJM, Normand E, Lodder JC, Rotaru DC (2016). Shisa6 traps AMPA receptors at postsynaptic sites and prevents their desensitization during synaptic activity. Nat Commun.

[84] Kim H-D, Wei J, Call T, Quintus NT, Summers AJ, Carotenuto S, Johnson R, Ma X, Xu C, Park JG (2021). Shisa6 mediates cell-type specific regulation of depression in the nucleus accumbens. Mol Psychiatry.

[85] Vialou V, Robison AJ, Laplant QC, Covington HE, Dietz DM, Ohnishi YN, Mouzon E, Rush AJ, Watts EL, Wallace DL (2010). ∆FosB in brain reward circuits mediates resilience to stress and antidepressant responses. Nat Neurosci.

[86] Iyilikci O, Balthazart J, Ball GF (2016). Medial preoptic regulation of the ventral Tegmental Area related to the control of Sociosexual Behaviors. eneuro.

[87] Groenewegen HJ, Russchen FT (1984). Organization of the efferent projections of the nucleus accumbens to pallidal, hypothalamic, and mesencephalic structures: a tracing and immunohistochemical study in the cat. J Comp Neurol.

[88] Heimer L, Zahm DS, Churchill L, Kalivas PW, Wohltmann C (1991). Specificity in the projection patterns of accumbal core and shell in the rat. Neuroscience.

[89] Salgado S, Kaplitt MG (2015). The Nucleus Accumbens: a Comprehensive Review. Stereotact Funct Neurosurg.

[90] Polzin BJ, Heimovics SA, Riters LV. Immunolabeling provides evidence for subregions in the songbird nucleus accumbens and suggests a context-dependent role in song in male European starlings (Sturnus vulgaris). *Brain, behavior and evolution* 2021. 10.1159/000521310 PMC8930704 34879382

[91] Moffitt JR, Bambah-Mukku D, Eichhorn SW, Vaughn E, Shekhar K, Perez JD, Rubinstein ND, Hao J, Regev A, Dulac C (2018). Molecular, spatial, and functional single-cell profiling of the hypothalamic preoptic region. Science.

[92] Validation of RNAseq. Experiments by qPCR? [http://bridgeslab.sph.umich.edu/posts/validation-of-rnaseq-experiments-by-qpcr].

[93] Hughes TR (2008). Validation’ in genome-scale research. J Biol.

[94] Fang Z, Cui X (2011). Design and validation issues in RNA-seq experiments. Brief Bioinform.

[95] Coenye T. Do results obtained with RNA-sequencing require independent verification? *Biofilm* 2021, 3. 10.1016/j.bioflm.2021.100043 PMC7823214 33665610

